# A Pilot Study of Circulating microRNA Expression in Newly Diagnosed Type 2 Diabetes Using a Pooled Sample Approach

**DOI:** 10.3390/clinpract16060100

**Published:** 2026-05-26

**Authors:** Loredana Deaconu, Romulus Zorin Timar, Cristiane Dragomir, Edward Seclaman, Anca Marcu, Diana Nitusca

**Affiliations:** 1 Second Department of Internal Medicine, “Victor Babes” University of Medicine and Pharmacy, 300041 Timisoara, Romania; loredana.deaconu@umft.ro (L.D.); timar.romulus@umft.ro (R.Z.T.); 2 Department of Diabetes, “Pius Brînzeu” Emergency County Hospital, 300723 Timisoara, Romania; 3 Center for Molecular Research in Nephrology and Vascular Disease, “Victor Babes” University of Medicine and Pharmacy, 300041 Timisoara, Romania; 4 Department of Biochemistry and Pharmacology, “Victor Babes” University of Medicine and Pharmacy, 300041 Timisoara, Romania; cristiane.dragomir@umft.ro (C.D.); eseclaman@umft.ro (E.S.); nitusca.diana@umft.ro (D.N.); 5 Center for Complex Networks Science, “Victor Babes” University of Medicine and Pharmacy, 300041 Timisoara, Romania

**Keywords:** miRNA, diabetes mellitus, pooled analysis, biomarkers

## Abstract

*Background and Objectives*: MicroRNAs (miRNAs) are small non-coding RNAs that regulate gene expression and have emerged as potential biomarkers in type 2 diabetes mellitus and its complications. This pilot exploratory study aimed to identify circulating miRNAs with differential expression in plasma from patients with newly diagnosed type 2 diabetes mellitus compared to age- and sex-matched healthy controls. *Materials and Methods*: Peripheral venous blood samples were collected from diabetic patients (*n* = 24) and controls (*n* = 12). Due to the exploratory nature of the study and limited sample material, samples were pooled within each group prior to plasma separation. Total RNA, including miRNAs, was extracted from plasma and analyzed using a high-throughput qPCR panel. Two normalization methods were applied to assess miRNA expression, and overlapping results were used for downstream analysis. Fold regulation was calculated using the 2^(−ΔCt) method. *Results*: A total of 33 and 42 miRNAs were identified as differentially expressed using the first and second normalization methods, respectively. Fourteen miRNAs were consistently downregulated across both methods. Several of these miRNAs, including hsa-miR-26a-5p, hsa-miR-146a-5p, hsa-miR-186-5p, hsa-miR-19a-3p, and hsa-miR-652-3p, have been previously associated with glucose metabolism, inflammation, and diabetic complications, such as retinopathy, neuropathy, and endothelial dysfunction. The pooling strategy enabled an efficient exploratory assessment of miRNA expression patterns while reducing inter-individual variability. *Conclusions*: This exploratory pilot study identifies a panel of circulating miRNAs with altered expression in pooled plasma samples from patients with newly diagnosed type 2 diabetes mellitus. These findings provide preliminary insights that warrant further validation in larger, individual-level studies to assess their diagnostic and prognostic potential.

## 1. Introduction

Diabetes mellitus represents a major global public health issue with increased morbidity and mortality caused by acute and chronic complications [1]. Among the various pathogenic factors involved, chronic hyperglycemia is recognized as the main driver of microvascular complications. The underlying pathophysiological mechanisms are complex, and over time, several interrelated biochemical pathways have been identified, including activation of the polyol pathway, formation of advanced glycation end products (AGEs), activation of protein kinase C (PKC) isoforms, and increased flux through the hexosamine pathway [2,3].

In recent years, studies have shown that miRNAs play a key role in the pathogenesis of type 2 diabetes and have received substantial attention as potential biomarkers in microvascular complications of this complex disease [4].

Experimental studies in animal models have demonstrated that modulation of specific miRNAs may exert protective effects, suggesting potential therapeutic applications. The discovery of a new set of miRNA biomarkers could help guide diagnostic and treatment decisions. However, new miRNA biomarkers need to be rigorously validated in independent, prospective, and adequately powered clinical trials before being implemented in clinical practice [5].

### 1.1. MicroRNAs

Ribonucleic acid (RNA) is a fundamental nucleic acid involved in gene expression regulation and multiple cellular processes, including transcriptional and post-transcriptional control. MicroRNAs are a class of non-coding RNAs that regulate gene expression and have an average length of 22 nucleotides. They were discovered in the 1990s in a nematode, *Caenorhabditis elegans* [6,7]. MicroRNAs are essential for normal development and are involved in a variety of biological processes. Aberrant expression of microRNAs [8], caused by internal (genes) or external (environment) factors [4], is associated with many pathological conditions [8]. In addition, these miRNAs are secreted into extracellular fluids; thus, extracellular miRNAs have been widely reported as potential biomarkers for a variety of diseases and as signaling molecules that mediate intercellular communication [8,9]. Furthermore, miRNAs have been reported to play an important role in regulating retinal pigment epithelium migration, proliferation, and apoptosis [10]. Furthermore, miRNA expression analysis by real-time RT-PCR is a technique with high sensitivity and specificity [11]. Therefore, miRNAs might be used as new biomarkers and therapeutic techniques for the diagnosis and prognosis of cancer and vascular disease.

Most miRNAs are transcribed by polymerase II from DNA sequences into primary miRNAs (pri-miRNAs) and are processed into precursor miRNAs (pre-miRNAs) by the complex formed by RNAase III Drosha together with its regulatory subunit DGCR8 [7,8,12]. Pre-miRNAs are transported into the cytoplasm by Exportin-5 to be cleaved into miRNA intermediates by Dicer RNAase III [7,12]. They then specifically interact with Argonaute proteins of the Ago subfamily and are incorporated into effector ribonucleoprotein complexes, called RISCs, that regulate the expression of target genes [7,13,14].

In most cases, miRNAs interact with the 3′-UTR regions of the target mRNA to suppress gene expression. However, interactions of miRNAs with other regions, including the 5′ UTR, have also been reported [8]. In addition, miRNAs have been shown to activate gene expression under certain conditions [15]. Recent studies have suggested that miRNAs are transported between different subcellular compartments to control translational and even transcriptional rates [16].

### 1.2. The Relationship Between miRNA and Diabetes Mellitus

In recent years, studies have shown that miRNAs play a key role in the pathogenesis of type 2 diabetes [4]. Pancreatic β-cells play a central role in glucose homeostasis through insulin secretion, and miRNAs associated with pancreatic β-cell dysfunction regulate cell survival, apoptosis, proliferation, differentiation, and insulin secretion [17]. Some miRNAs promote β-cell proliferation, whereas others exert inhibitory effects [13]. One of the most important miRNA regulators is miR-375, which is highly expressed in both human and mouse pancreatic β-cells and is indispensable in maintaining normal pancreatic β-cell mass [13,17].

Insulin resistance is a deficient cellular response to insulin and the inability of normal insulin levels to maintain normal glucose homeostasis, which is an important feature in the pathogenesis of type 2 diabetes mellitus. In this process, the insulin signaling pathway plays a central role [17]. Thus, miRNAs can regulate insulin response in target tissues. For example, miR-29a and miR-29b primarily regulate the insulin signaling pathway by inhibiting proteins that enhance insulin signaling [4]. MiR-126 can increase insulin resistance by inhibiting IRS1 [17]. In addition, miRNAs can also directly regulate glucose levels in various cells (e.g., miR-223 can regulate glucose uptake by inhibiting GLUT4 in muscle tissue) [4,17]. MiR-33a and miR-33b can regulate the insulin pathway via IRS2, SIRT6, and AMPKα1 [4]. MiR-130a and miR-204 can improve glucose tolerance by inhibiting GRB10 and GLP1R, respectively. miR-378 and miR-93 lead to insulin resistance by targeting P110a and SIRT7, respectively [4,18].

In serum and plasma, miRNAs were initially found in exocytosis vesicles and in particles secreted by donor cells [19]. Subsequent studies showed that miRNAs also existed in apolipoproteins. For example, miR-126 is associated with type 2 diabetes, and miR-486, miR-146b, miR-424, and miR-15b are increased in circulating samples derived from patients with diagnosed type 2 diabetes [4].

The involvement of miRNAs in regulating insulin signaling pathways and glucose homeostasis underscores their important role in the pathogenesis of type 2 diabetes. The identification of circulating miRNAs in serum and plasma not only provides promising non-invasive biomarkers for this disease but also suggests a potential role in intercellular communication between donor and target tissues. Advances in understanding miRNA metabolism and function have revealed potential therapeutic targets in type 2 diabetes, including miR-33a and miR-33b. Additionally, miR-103 and miR-107 have been proposed as potential candidates for pharmacological intervention [4].

Pancreatic miRNAs act through a diverse set of pathways that regulate β-cell development and biological function. Disruption of miRNA expression profiles in β-cells has been shown to elucidate much of the pathology associated with type 1 and type 2 diabetes. In addition to the role of applying expanded miRNA profiles to predict diabetes onset, regulation of pancreatic miRNA targets may help develop novel clinical therapies that modulate the expression and activity of these miRNAs, restoring normal glucose homeostasis and β-cell function [14].

Several studies have identified altered circulating miRNA expression profiles in patients with prediabetes and newly diagnosed T2DM. Reduced circulating levels of miR-126, one of the most extensively investigated endothelial-associated miRNAs, have been associated with impaired glucose tolerance and newly diagnosed T2DM [20]. Similarly, dysregulation of miR-146a, miR-29a, miR-375, and other miRNAs implicated in insulin signaling, inflammation, and β-cell function has been reported in early stages of the disease [21,22,23]. These findings support the potential utility of circulating miRNAs as minimally invasive biomarkers for early detection and metabolic characterization of T2DM.

However, despite the growing number of studies investigating circulating miRNAs in diabetes, considerable heterogeneity remains across published data. Variability in study design, patient selection, sample processing, RNA extraction protocols, profiling platforms, and normalization methods substantially affects the reproducibility and comparability of reported miRNA signatures [24,25]. In particular, normalization of circulating miRNA data remains a major methodological challenge, as no universally accepted endogenous control has been established for plasma or serum samples [25].

In this regard, the objective of this case–control pilot study was to investigate differential circulating microRNA expression profiles in pooled plasma samples derived from patients with diagnosed type 2 diabetes mellitus compared to healthy controls, to identify candidate miRNAs that may serve as potential diagnostic biomarkers and provide insight into the molecular mechanisms underlying the disease. Despite their exploratory nature, our results support the methodological feasibility of this strategy and lay the groundwork for future large-scale investigations to integrate miRNA expression profiles into standard care for type 2 diabetes.

Although numerous studies have investigated circulating miRNAs in type 2 diabetes mellitus, relatively limited data are available regarding newly diagnosed patients before prolonged antidiabetic treatment exposure and advanced chronic complications. Furthermore, few exploratory studies have evaluated reproducible circulating miRNA patterns across multiple normalization strategies in pooled plasma samples. Therefore, the present pilot study aimed to identify candidate circulating miRNAs consistently associated with newly diagnosed type 2 diabetes mellitus using a high-throughput qPCR platform combined with complementary bioinformatic analyses.

## 2. Materials and Methods

### 2.1. Research Design and Study Cohort

This case–control pilot study enrolled 36 adult participants who were recruited voluntarily from the Emergency County Hospital “Pius Brînzeu” in Timișoara, Romania. The study group consisted of 24 patients with newly diagnosed type 2 diabetes mellitus, while the control group included 12 age-matched healthy individuals without a history of diabetes or other major chronic diseases.

The inclusion criteria were age over 18 years and a recent diagnosis of type 2 diabetes mellitus. Pregnant women and individuals with severe acute or chronic comorbidities were excluded. All participants provided written informed consent before their enrollment in the study and prior to blood sample collection. Clinical and demographic characteristics of the patients included in our study were extracted from medical records and obtained as part of routine laboratory analyses performed within the hospital laboratory.

The research protocol was approved by the Ethics Committee of the Emergency County Hospital “Pius Brînzeu” in Timișoara, Romania (approval no. 283/2 March 2022). The study was conducted in accordance with the ethical principles of the Declaration of Helsinki (2013 revision), and all personal data were handled confidentially in compliance with the General Data Protection Regulation (GDPR) standards.

### 2.2. Specimen Collection and Processing

Venous blood samples were collected in EDTA-treated tubes under standardized conditions. The samples were immediately transported on ice to the laboratory of the Discipline of Biochemistry from “Victor Babeș” University of Medicine and Pharmacy, Timișoara, Romania (UMFVBT), where plasma was separated by centrifugation at 2000× *g* for 15 min. The resulting plasma samples were subsequently aliquoted and stored at −80 °C until further analysis.

Total RNA, including miRNA fractions, was isolated from pooled plasma using the miRNeasy Mini Kit (Qiagen, Hilden, Germany), in accordance with the manufacturer’s instructions. Each pool consisted of 8 individual samples from the patient group (P) and 4 from the control group (C), providing a representative overview of collective miRNA expression in each cohort. This pooling strategy is particularly useful in pilot studies, as it enables initial broad screening of numerous miRNAs while conserving reagents and minimizing technical variability. For this study, three patient pools (P1, P2, P3) and three control pools (C1, C2, C3) were generated to maximize the likelihood of identifying miRNAs consistently associated with type 2 diabetes. Due to the exploratory nature of the study and limited biological material, plasma samples were pooled before RNA extraction and qPCR analysis. Three pools from patients with type 2 diabetes mellitus and three pools from healthy controls were generated. Each diabetic pool contained plasma derived from 8 individual participants, while each control pool contained plasma from 4 participants. In the present exploratory design, pooled samples represented the experimental and analytical units.

Each patient pool (P) reflects the biological state of 8 individuals recently diagnosed with type 2 diabetes, while each control pool (C) represents 4 age- and sex-matched healthy participants. Pools were randomly assembled while respecting the biological sex distribution within each group. Although pooling can mask individual variability, it provides a practical approach to detect overall trends in miRNA expression, particularly in an exploratory context.

Labeling and chain-of-custody procedures were implemented to ensure sample traceability and integrity throughout all processing stages. The purified RNA samples were stored at −80 °C until reverse transcription and subsequent quantitative polymerase chain reaction (qPCR) analysis.

### 2.3. Analysis of Plasma miRNA Expression

Total RNA was reverse-transcribed into complementary DNA (cDNA) using a miRNA-specific kit (Qiagen, Hilden, Germany), and the resulting cDNA was analyzed by quantitative real-time PCR (qPCR) using a high-throughput human miRNA panel (Human Serum/Plasma Focus miRCURY LNA miRNA Focus PCR Panel–Qiagen GeneGlobe ID: YAHS-106Y). Quality controls included UniSP2, UniSP4, UniSP5, and an external spike-in control (cel-miR-39) to monitor amplification efficiency. UniSP3 was used as an inter-plate calibrator. Threshold cycle (Ct) values were normalized using various reference strategies recommended by the Qiagen platform. Relative miRNA expression was calculated using the 2^(−ΔCt) method. Rigorous quality-control procedures were applied to ensure inter-plate consistency, and any outlier measurements were carefully reviewed prior to inclusion in the final analysis. Fold regulation values were derived from fold change calculations according to Qiagen GeneGlobe conventions, where fold changes below 1 were transformed into negative inverse values to indicate downregulation.

The Qiagen miRCURY LNA miRNA Serum/Plasma Focus PCR Panel was used for profiling analysis. miRNAs with Ct values > 35 or with inconsistent amplification profiles were excluded from downstream analyses. Hemolysis-related quality assessment was performed using spectrophotometric measurements at 414 nm.

### 2.4. Statistical Analysis

Data analysis was performed using SPSS Statistics v.27 (IBM Corp., Armonk, NY, USA). As most miRNA expression data deviated from normality, non-parametric statistical methods, primarily the Mann–Whitney U test, were used to compare expression levels between patient and control groups. Normalization was performed via two different methods provided by the Qiagen GeneGlobe platform (https://geneglobe.qiagen.com/us/analyze, accessed on 17 February 2026), global Ct mean of expressed miRNAs, and geNorm normalization using the entire miRNA panel.

Statistical analysis of the characteristics groups was performed with MedCalc^®^ Statistical Software v.23.3.7. Normally distributed variables are presented as the mean ± standard deviation (SD). In contrast, non-normally distributed variables are presented as the median and interquartile range (IQR). Unpaired *t*-tests or Mann–Whitney U tests were used to evaluate differences between participants’ sex depending on variable distribution. Statistical significance was considered at *p* < 0.05. This statistical analysis refers only to baseline characteristics, clinical, and demographic data of the individual patients.

Given the pooled-sample exploratory design, statistical analyses were interpreted descriptively and exploratory rather than inferentially at the individual participant level. Differential expression patterns were evaluated between pooled samples generated from diabetic and control groups. *p*-values were reported as exploratory indicators of expression differences and should not be interpreted as definitive evidence of statistical significance for individual-level biological variation.

Given the pilot nature of the study, the limited number of pooled observations, and the objective of identifying candidate miRNAs for future validation, we elected to retain the exploratory screening approach while explicitly acknowledging the increased risk of false-positive findings.

## 3. Results

### 3.1. Clinical and Demographic Characteristics of the Subjects

A total of 36 individuals were included in our study (Table 1) with a median age of 53 years (IQR 49–57). Among them, 52.8% are men (median age 51 years [IQR 49–54]), and 47.2% are women (median age 56 years [IQR 52–61.25]), with a two-tailed *p*-value of 0.0361. The control group (*n* = 12), consisting of 6 men and 6 women, does not differ significantly from the test group in age (median 51 years [IQR 49–54]) or gender distribution. It is important to note that the controls were healthy and did not receive any medical treatment at the start of the study.

Clinical and demographic characteristics of the patients included in our study are presented in Table 2. The study included 24 patients with a mean age of 53.5 ± 8.5. The median of HbA1c is >9.03 (IQR 6.74–11.05). The patients were not diagnosed with prediabetes before, and they were not taking any medications for diabetes. Regarding LDLc (102.5 mg/dL, IQR 78.5–152.5) and TG (206.95 ± 139.87), we note that 41.67% of patients were on statin therapy before the start of the study. Also, all patients underwent fibroscan (median 5.75 kPa [IQR 4.75–6.65]) and CAP (median 325 dB/m [IQR 272–353]). The modified CAP classifies patients into different grades of steatosis. Regarding inflammation, we measured IL6 and CRP and observed that 33% of patients have IL6 > 7 pg/mL (5.767 ± 5.088) and 41.66% have CRP > 10 mg/L (13.31 ± 11.22), indicating a general inflammatory state.

Regarding the sex distribution of the study (Table 3), no significant difference was observed in age (*p* = 0.195), but significant differences were observed in height (*p* = 0.0001) and eGFR (*p* = 0.027). Most patients were obese, with a BMI of 32.41 kg/m^2^ (IQR 29.46–40.34) in men and 37.83 kg/m^2^ (IQR 29.16–42.09) in women, with no significant difference between the two groups (*p* = 0.69). We observed that women had significantly lower uric acid levels (4.9 mg/dL [IQR 3.92–5.57] vs. 6.5 mg/dL [IQR 5.83–7.06]; *p* = 0.03), probably due to the protective effects of estrogen.

For other chronic complications (Table 4), fundoscopic data were extracted from medical records, and none showed retinopathy. Also, we measured the ankle-brachial index (ABI), and only 1 patient had peripheral artery disease (ABI < 0.9). Regarding the macrovascular complication (stroke, coronary syndrome), we performed the anamnesis, physical examination, personal history, and EKG, and the results indicate that 25% of them had coronary diseases, and 4.2% had cerebrovascular disease. Furthermore, 58.3% of the study patients suffer from diabetic neuropathy, and 79.2% present hypertension.

### 3.2. miRNA Analysis

Due to the pooling strategy, the statistical analyses reflect differences within the pooled samples rather than between independent biological replicates. Therefore, *p*-values are presented as exploratory measures of differential expression and should not be interpreted as definitive evidence of significance at the individual participant levels.

Using the first normalization method provided by the Qiagen GeneGlobe platform (Global Ct mean of expressed miRNAs), 33 miRNAs showed altered expression patterns in pooled plasma samples, relative to the control group, of which 29 were downregulated, and 4 were upregulated (Table 5).

As per the second normalization method (geNorm–entire miRNA panel), a total of 42 miRNAs demonstrated altered expression profiles, and from them, only 3 (hsa-miR-155-5p, hsa-miR-136-5p, and hsa-miR-136-3p) showed an upregulated expression, and the other 39 miRNAs were down-regulated, compared to the control group (Table 6).

**Figure 1:**
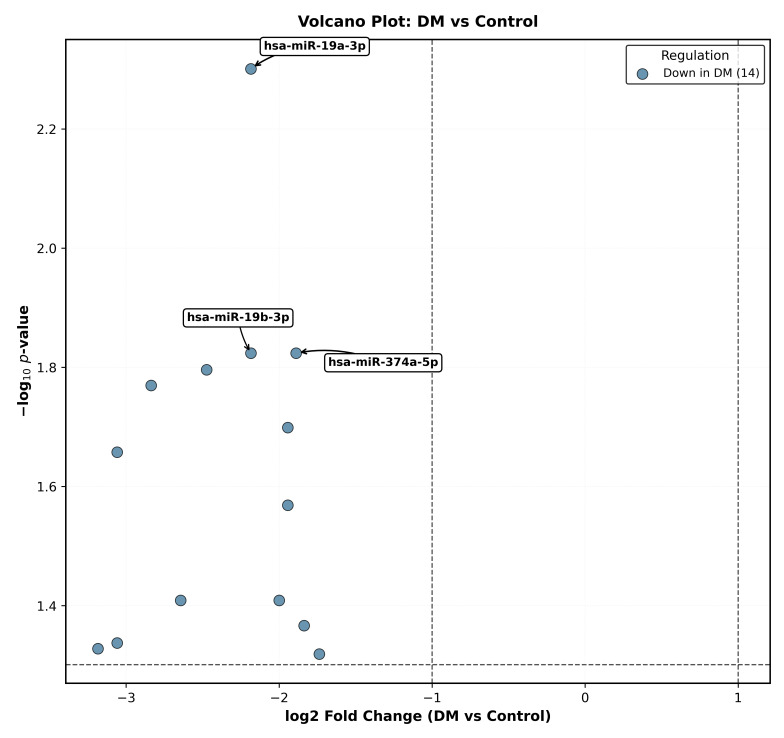
Volcano plot of differentially expressed miRNAs in DM. The x-axis shows log2 fold change between DM and Control groups. The y-axis shows −log10 *p*-value. Dashed lines represent significance thresholds: *p*-value < 0.05 and |fold change| > 2. Blue points indicate significantly downregulated miRNAs in DM (*n* = 14). The top 3 most significant miRNAs are labeled. All tested miRNAs showed significant downregulation in DM patients compared to healthy controls.

Overlapping dysregulated miRNAs (*n* = 14) identified by both normalization methods in plasma from type 2 diabetes patients compared with controls are shown in Table 7 and Figure 1.

Because of the exploratory pilot design, limited number of pooled observations, and large number of analyzed targets, these findings should be interpreted as exploratory expression signals that require validation in larger independent cohorts.

### 3.3. Gene Analysis

Furthermore, we have identified target genes (target score ≥ 80) for each miRNA in miRDB (https://mirdb.org/, accessed on 17 February 2026). From this analysis, we selected the genes associated with diabetes mellitus, obesity, dyslipidemia, inflammation, and steatosis with a relevance score ≥ 10, based on data from GeneCards (https://www.genecards.org/, accessed on 17 February 2026) (Table 8).

Next, we searched for the same genes with strong evidence in miRTarBase (https://mirtarbase.cuhk.edu.cn/~miRTarBase/miRTarBase_2025, accessed on 17 February 2026) (Table 9). In our study, all selected miRNAs were downregulated, associated with increased expression of target genes, directly or indirectly, that contribute to insulin resistance, dysregulated glucose metabolism, and chronic inflammation due to the molecular alterations.

## 4. Discussion

The present study only reports the observed circulating miRNA expression patterns in pooled plasma samples and does not directly assess tissue expression, mechanistic activity, or causal biological effects. Associations with metabolic, inflammatory, and diabetes-related pathways are now explicitly presented as observations supported by prior literature and bioinformatic analyses rather than direct conclusions derived from our dataset.

In this pilot study, we identified 14 miRNAs that were consistently downregulated across both normalization methods, representing the most robust and reproducible findings. The overlap between the two analytical approaches strengthens the validity of these miRNAs as potential biomarkers in type 2 diabetes mellitus. Consistently downregulated miRNAs may reflect underlying alterations in gene regulatory networks associated with glucose metabolism, insulin signaling, or β-cell function. Their reproducibility across different normalization strategies highlights their potential as reliable indicators of disease-associated molecular changes, warranting further validation in larger cohorts and functional studies to explore their mechanistic roles in the pathophysiology of type 2 diabetes.

Several of the downregulated miRNAs in our cohort have been implicated in type 2 diabetes and its complications. For instance, miR-652-3p was reported by Villard et al. in a meta-analysis to be decreased in patients with type 2 diabetes mellitus, supporting its potential as a disease-associated biomarker [26]. Similarly, miR-26a-5p has been shown to target USP14 and inactivate the NF-κB signaling pathway in mice, providing a protective effect against inflammation and oxidative stress in diabetic retinopathy [27]. Clinical studies also indicate that miR-26a-5p and miR-146b-5p are downregulated in patients with proliferative and non-proliferative diabetic retinopathy, suggesting that decreased circulating levels may reflect early retinal neurodegeneration [28]. Although our cohort did not include patients with diabetic retinopathy, the downregulation of miR-26a-5p could indicate a heightened risk for future retinal complications.

Other miRNAs identified in our study have been linked to metabolic and inflammatory pathways. miR-222-3p was reported to be decreased in patients with type 2 diabetes mellitus and negatively correlated with OGTT and HbA1c levels, while miR-18b-5p was associated with lipid profile parameters [27,29]. miR-146a-5p, known to regulate inflammatory responses by targeting IRAK1 and NFT5, was downregulated in our cohort, corresponding with moderately elevated IL-6 (5.77 ± 5.09) and CRP (13.31 ± 11.22) levels, suggesting a low-grade inflammatory state. Previous studies also demonstrated that reduced glomerular expression of miR-146a correlates with albuminuria in patients with diabetes [30,31].

Additional miRNAs in our study, such as miR-186-5p, miR-19a-3p, miR-19b-3p, miR-140-3p, and miR-142-3p, have been associated with diabetic neuropathy, cardiomyopathy, endothelial dysfunction, and renal injury, highlighting their potential relevance to multiple organ-specific complications [32,33,34,35,36,37].

### Limitations

Obesity and type 2 diabetes mellitus are closely interconnected, and the inclusion of an additional obese non-diabetic control group would provide further insight into the specific contribution of obesity-related metabolic alterations to circulating miRNA expression. Future studies including obese non-diabetic participants and larger cohorts will be necessary to better distinguish obesity-related from diabetes-specific miRNA signatures.

It is important to note that our study employed a pooling strategy, with 8 patient samples and 4 control samples per pool, to provide a representative snapshot of collective miRNA expression. While pooling enables broad exploratory screening, reduces reagent consumption, and minimizes technical variability, it also introduces inherent limitations. Individual-level variability is not captured, preventing the assessment of associations between miRNA expression and patient-specific individual clinical or biological parameters. Consequently, the variability reflected in the results corresponds to differences between pools rather than between individual participants. Although individual Ct values were retained and examined for potential outliers, this approach does not fully account for inter-individual heterogeneity.

The study uses pooled plasma samples as analytical units. Although pooling represents a practical and cost-effective strategy for exploratory high-throughput screening, this approach masks inter-individual variability and limits the ability to correlate miRNA expression patterns with participant-specific clinical or biological parameters. Consequently, the observed expression profiles reflect pooled biological signals rather than independent individual-level measurements.

Additional limitations include a relatively small cohort, the exploratory nature of the pilot design, and the lack of a direct correlation between miRNA expression and individual clinical outcomes. The relatively small cohort size further limits the generalizability and statistical robustness of the findings. Therefore, the identified circulating miRNA patterns should be considered preliminary and hypothesis-generating until validated in larger prospective studies using individual-level analyses. Despite these limitations, high-throughput qPCR panels yielded reproducible measurements, and the identified miRNAs showed consistent differential expression patterns compared with controls, supporting their potential relevance for further validation in larger individual-level studies.

## 5. Conclusions

In summary, this exploratory pilot study describes altered circulating miRNA expression patterns observed in pooled plasma samples from patients with newly diagnosed type 2 diabetes mellitus compared with healthy controls. Several identified miRNAs have previously been associated with metabolic and inflammatory pathways relevant to diabetes mellitus. However, given the pooled-sample design, limited cohort size, and exploratory statistical framework, these findings should be considered preliminary and hypothesis-generating. Larger individual-level validation studies are required to determine their biological and clinical relevance.

## Figures and Tables

**Table 1:** Baseline characteristics of the study participants (*n* = 36).

Variable	All (Median + IQR)	Men (Median + IQR)	Women (Median + IQR)	DM (Median + IQR)	Controls (Median + IQR)
Age (years)	53 (49–57) *n* = 36	51 (49–54) *n* = 19	56 (52–61.25) *n* = 17	54.5 (50–59.5) *n* = 24	51 (49–54) *n* = 12

Values are expressed as the median and IQR. The Mann–Whitney test was performed. Abbreviations: IQR = interquartile range; DM = diabetes mellitus.

**Table 2:** Clinical and demographic characteristics of the study patients (*n* = 24).

Variable	Value
Age (years)	53.5 ± 8.5
Weight (kg)	101.5 (82–112.5)
Height (m)	1.695 (1.635–1.765)
BMI (kg/m^2^)	35.19 (29.375–40.885)
WC (cm)	118.5 (106–121.5)
Glycemia (mg/dL)	198.58 ± 109.51
HbA1c (%)	9.03 (6.74–11.05)
TC (mg/dL)	181 (142–252.5)
LDLc (mg/dL)	102.5 (78.5–152.5)
TG (mg/dL)	206.95 ± 139.87
HDLc (mg/dL)	41.5 (34.5–47.5)
nonHDLc (mg/dL)	141.5 (100.5–204)
SCr (mg/dL)	0.78 (0.68–0.90)
eGFR (ml/min)	96.6 ± 18.45
ACR (mg/g)	40.645 ± 66.886
ALT (U/L)	49.25 ± 24.12
CAP (dB/m)	325 (272–353)
Fibroscan (kPa)	5.75 (4.75–6.65)
IL6 (pg/mL)	5.767 ± 5.088
CRP (mg/L)	13.31 ± 11.22
Uric acid (mg/dL)	5.9 (4.9–6.85)

Abbreviations: BMI, body mass index; WC, waist circumference; TC, total cholesterol; LDLc, low-density lipoprotein; TG, triglyceride; HDLc, high-density lipoprotein; SCr, serum creatinine; eGFR, estimated glomerular filtration rate; ACR, albumin-creatinine ratio; ALT, alanine aminotransferase; CAP, controlled attenuation parameter; IL6, interleukin 6; CRP, C-reactive protein. Values are expressed as the mean ± SD for normally distributed variables and as the median (IQR) for non-normally distributed variables.

**Table 3:** Baseline characteristics by sex.

Variable	Men	Women	*p*-Value
Age (years) ^a^	51.38 *n* = 13	56.00 *n* = 11	0.1958 ^ns^
Weight (kg) ^b^	104.00 89.50 to 119.50	100.00 81.25 to 108.50	0.4005 ^ns^
Height (m) ^b^	1.75 1.71 to 1.79	1.63 1.61 to 1.65	0.0001 **
BMI (kg/m^2^) ^b^	32.41 29.46 to 40.34	37.83 29.16 to 42.09	0.6905 ^ns^
WC (cm) ^b^	118.00 104.50 to 123.25	119.00 106.75 to 121.75	0.8162 ^ns^
Glycemia (mg/dL) ^a^	184.69 *n* = 13	215.00 *n* = 11	0.5115 ^ns^
HbA1c (%) ^b^	9.00 6.50 to 10.53	9.06 6.85 to 12.21	0.2705 ^ns^
TC (mg/dL) ^b^	193.00 146.00 to 221.50	160.00 141.00 to 252.75	0.9077 ^ns^
LDLc (mg/dL) ^b^	103.00 84.25 to 126.00	98.00 59.20 to 168.00	0.6637 ^ns^
TG (mg/dL) ^a^	207.38 *n* = 13	206.45 *n* = 11	0.9875 ^ns^
HDLc (mg/dL) ^b^	40.00 33.75 to 46.00	45.00 36.25 to 48.75	0.4004 ^ns^
nonHDLc (mg/dL) ^b^	154.00 105.25 to 188.25	125.00 96.50 to 204.50	0.8848 ^ns^
SCr (mg/dL) ^b^	0.80 0.70 to 0.90	0.70 0.66 to 0.94	0.3993 ^ns^
eGFR (ml/min) ^a^	104.09 *n* = 13	87.75 *n* = 11	0.0270 *
ACR (mg/g) ^a^	19.11 *n* = 12	64.14 *n* = 11	0.1081 ^ns^
ALT (U/L) ^a^	56.31 *n* = 13	40.91 *n* = 11	0.1214 ^ns^
CAP (dB/m) ^b^	338.00 263.75 to 380.25	312.00 278.50 to 348.75	0.8620 ^ns^
Fibroscan (kPa) ^b^	5.70 4.57 to 6.98	6.00 4.98 to 6.28	0.9307 ^ns^
IL6 (pg/mL) ^a^	4.47 *n* = 13	7.30 *n* = 11	0.1815 ^ns^
CRP (mg/L) ^a^	11.32 *n* = 13	15.66 *n* = 11	0.3562 ^ns^
Uric acid (mg/dL) ^b^	6.50 5.83 to 7.05	4.90 3.92 to 5.75	0.0319 *

^a^ T-test; ^b^ Mann–Whitney test; Abbreviations: BMI, body mass index; WC, waist circumference; TC, total cholesterol; LDLc, low-density lipoprotein; TG, triglyceride; HDLc, high-density lipoprotein; SCr, serum creatinine; eGFR, estimated glomerular filtration rate; ACR, albumin-creatinine ratio; ALT, alanine aminotransferase; CAP, controlled attenuation parameter; IL6, interleukin 6; CRP, C-reactive protein; ns = not significant *p* ≥ 0.5, * = statistically significant *p* < 0.05, ** = very highly statistically significant *p* < 0.001. Values are expressed as the mean ± SD for normally distributed variables and as the median (IQR) for non-normally distributed variables.

**Table 4:** Frequency of complications and comorbidities.

Variable	With (%)	Without (%)
Diabetic neuropathy	58.3 (*n* = 14)	41.7 (*n* = 10)
Coronary disease	25 (*n* = 6)	75 (*n* = 18)
Cerebrovascular disease	4.2 (*n* = 1)	95.8 (*n* = 23)
Hypertension	79.2 (*n* = 19)	20.8 (*n* = 5)

**Table 5:** Plasma miRNAs exhibiting differential expression in type 2 diabetes patients relative to controls (using the first normalization method).

MiRNA	*p*-Value	Fold Change	Fold Regulation
hsa-miR-652-3p	0.020	0.26	−3.91
hsa-miR-106a-5p	0.023	0.39	−2.55
hsa-miR-26a-5p	0.016	0.18	−5.65
hsa-miR-222-3p	0.047	0.11	−8.90
hsa-miR-152-3p	0.025	0.29	−3.44
hsa-miR-146a-5p	0.046	0.12	−8.13
hsa-miR-148b-3p	0.008	0.25	−3.99
hsa-miR-20a-5p	0.020	0.43	−2.32
hsa-miR-17-5p	0.016	0.45	−2.22
hsa-miR-186-5p	0.043	0.28	−3.58
hsa-miR-142-3p	0.039	0.25	−4.04
hsa-miR-126-5p	0.024	0.55	−1.81
hsa-miR-19a-3p	0.005	0.22	−4.57
hsa-miR-144-3p	0.048	0.27	−3.67
hsa-miR-126-3p	0.025	0.36	−2.78
hsa-miR-18b-5p	0.048	0.30	−3.30
hsa-miR-19b-3p	0.015	0.22	−4.55
hsa-miR-502-3p	0.011	0.51	−1.96
hsa-miR-140-3p	0.022	0.12	−8.48
hsa-miR-532-3p	0.039	0.16	−6.20
hsa-miR-15a-5p	0.043	0.49	−2.05
hsa-miR-532-5p	0.044	0.39	−2.56
hsa-miR-660-5p	0.002	0.35	−2.83
hsa-miR-497-5p	0.009	5.53	5.53
hsa-miR-18a-5p	0.045	0.31	−3.18
hsa-miR-29b-3p	0.017	0.14	−7.02
hsa-miR-1260a	0.019	3.36	3.36
hsa-miR-100-5p	0.0004	2.43	2.43
hsa-miR-146b-5p	0.027	0.26	−3.88
hsa-miR-378a-3p	0.003	0.32	−3.09
hsa-miR-374a-5p	0.015	0.27	−3.74
hsa-miR-483-5p	0.025	10.78	10.78
hsa-miR-21-5p	0.025	0.41	−2.44

**Table 6:** Plasma miRNAs exhibiting differential expression in type 2 diabetes patients relative to controls (using the second normalization method).

MiRNA	*p*-Value	Fold Change	Fold Regulation
hsa-miR-652-3p	0.024	0.23	−4.32
hsa-miR-374b-5p	0.014	0.28	−3.55
hsa-miR-93-5p	0.012	0.28	−3.55
hsa-miR-484	0.003	0.41	−2.44
hsa-miR-26a-5p	0.014	0.16	−6.23
hsa-miR-222-3p	0.042	0.10	−9.82
hsa-miR-16-5p	0.040	0.25	−3.93
hsa-miR-30c-5p	0.046	0.20	−5.11
hsa-miR-146a-5p	0.048	0.11	−8.96
hsa-miR-107	0.019	0.10	−9.84
hsa-miR-186-5p	0.002	0.25	−3.95
hsa-miR-320b	0.036	0.43	−2.31
hsa-miR-301a-3p	0.021	0.31	−3.26
hsa-miR-151a-5p	0.016	0.25	−4.01
hsa-miR-320a-3p	0.033	0.40	−2.48
hsa-miR-103a-3p	0.021	0.11	−8.90
hsa-miR-142-3p	0.008	0.22	−4.46
hsa-miR-19a-3p	0.042	0.20	−5.04
hsa-miR-195-5p	0.022	13.25	13.25
hsa-miR-18b-5p	0.037	0.27	−3.64
hsa-miR-320d	0.015	0.40	−2.53
hsa-miR-19b-3p	0.018	0.20	−5.03
hsa-miR-155-5p	0.035	2.74	2.74
hsa-miR-140-3p	0.0004	0.11	−9.36
hsa-miR-92b-3p	0.007	2.06	2.06
hsa-let-7d-5p	0.013	0.25	−3.92
hsa-miR-532-3p	0.001	0.15	−6.84
hsa-miR-320c	0.007	0.36	−2.77
hsa-miR-130a-3p	0.003	0.22	−4.53
hsa-let-7c-5p	0.013	0.36	−2.74
hsa-miR-29b-3p	0.049	0.13	−7.74
hsa-miR-136-5p	0.015	1.91	1.91
hsa-miR-146b-5p	0.008	0.23	−4.28
hsa-miR-339-5p	0.049	0.13	−7.81
hsa-miR-425-5p	0.042	0.26	−3.83
hsa-miR-16-2-3p	0.012	0.44	−2.26
hsa-miR-130b-3p	0.010	0.26	−3.79
hsa-miR-363-3p	0.045	0.24	−4.15
hsa-miR-374a-5p	0.034	0.24	−4.12
hsa-miR-151a-3p	0.006	0.27	−3.68
hsa-miR-136-3p	0.044	6.83	6.83
hsa-miR-15b-5p	0.002	0.21	−4.73

**Table 7:** Commonly dysregulated miRNAs were detected across both normalization methods.

miRNA	Ct Average ± SD		*p*-Value (<0.05)	Fold Change	Fold Regulation
	DM	Controls			
hsa-miR-652-3p	33.55 ± 1.85	27.22 ± 0.91	0.020	0.26	−3.91
hsa-miR-26a-5p	32.46 ± 1.39	25.60 ± 0.78	0.016	0.18	−5.65
hsa-miR-222-3p	34.06 ± 1.11	26.55 ± 0.85	0.047	0.11	−8.90
hsa-miR-146a-5p	33.70 ± 1.55	26.32 ± 0.96	0.046	0.12	−8.13
hsa-miR-186-5p	34.47 ± 0.89	28.26 ± 0.75	0.043	0.28	−3.58
hsa-miR-142-3p	31.10 ± 1.55	24.73 ± 0.84	0.039	0.25	−4.04
hsa-miR-19a-3p	27.94 ± 0.43	21.38 ± 0.50	0.005	0.22	−4.57
hsa-miR-18b-5p	34.59 ± 0.71	28.50 ± 0.53	0.048	0.30	−3.30
hsa-miR-19b-3p	28.30 ± 0.37	21.75 ± 0.51	0.015	0.22	−4.55
hsa-miR-140-3p	32.20 ± 0.52	24.76 ± 0.79	0.022	0.12	−8.48
hsa-miR-532-3p	34.82 ± 0.16	27.82 ± 0.73	0.039	0.16	−6.20
hsa-miR-29b-3p	34.31 ± 0.60	27.14 ± 0.53	0.017	0.14	−7.02
hsa-miR-146b-5p	34.69 ± 0.54	28.37 ± 0.60	0.027	0.26	−3.88
hsa-miR-374a-5p	34.36 ± 0.78	28.09 ± 0.50	0.015	0.27	−3.74

Abbreviations: miR—microRNA, Ct—cycle threshold, SD—standard deviation.

**Table 8:** Gene analysis.

miRNA	Pathology	Common Genes
miR-652-3p	Diabetes	ISL1
miR-26a-5p	Diabetes	PTEN, ADM, GSK3B, PTGS2, EP300, PPP1R15B, SLC5A1, FA2H, HGF, SLC19A2, TBC1D4, MTTP, HMGA1, ESR1, SELP, ATM, NAMPT
	Obesity	PTEN, ABHD5, FBXO11, EP300, SLC5A1, PHF6, MTTP, ESR1, ACADM, NAMPT, CREBBP, BBS7
	Dyslipidemia	MTTP
	Inflammation	PTEN, ADAM17, PTGS2, SELP
	Steatosis	MTTP
miR-222-3p	Diabetes	PIK3R1, CXCL12, KDR, ESR1, MIA3, FXN, DNAJC6, CASR
	Obesity	POGZ, MRAP2, PIK3R1, ESR1, TUB, SLC6A4
	Inflammation	CXCL12
miR-146a-5p	Diabetes	APPL1, HIPK3, CCK, CCL5, CYP27B1
	Obesity	CCK, CYP27B1, HDAC8
	Inflammation	IRAK1, CCL5
miR-186-5p	Diabetes	PIK3CA, SCN9A, PRKAA2, SUMO4, PIK3CG, PTH, TRMT10A, LPIN1, SIRT1, MAPK1, VEGFA, ANGPT2, PCSK2
	Obesity	NEGR1, VPS13B, PIK3CA, PHIP, PDE4D, PHF6, NSD1, LPIN1, SIRT1
	Inflammation	PIK3CG, GJA1, XIAP, SIRT1, VEGFA
miR-142-3p	Diabetes	GHR, TAB2, PGM1, NR3C1, ITPR3
	Obesity	CLOCK, GHR, NR3C1, SH2B1
	Inflammation	IRAK1
miR-19a-3p	Diabetes	ESR1, PIK3CA, SGK1, ABCA1, IGFBP3, LDLR, PON2, TNFAIP3, ADIPOR2, NEUROD1, F3, IGF1, PTEN, SERPINE1, PPARA, SOCS1, SOCS3, MAPK1, JAZF1, MAPK8, PRKAA1, APPL1, HIPK3, EYA1, CBLB, ITCH, UCP3, GFPT1
	Obesity	ESR1, CLOCK, PIK3CA, ABCA1, IGFBP3, LDLR, HPRT1, ADIPOR2, CAST, ABHD5, IGF1, PTEN, TUB, SERPINE1, PPARA, SOCS3, UCP3, GFPT1
	Dyslipidemia	ABCA1, LDLR
	Inflammation	GJA1, TNFAIP3, PTEN, SERPINE1, SOCS1, MAPK14, TBK1, TEK, TNIP1
miR-18b-5p	Diabetes	HIF1A, TNFAIP3, ESR1, CCN2, ATM
	Obesity	PDE4D, ESR1
	Inflammation	HIF1A, TNFAIP3
miR-19b-3p	Diabetes	ESR1, PIK3CA, SGK1, ABCA1, IGFBP3, LDLR, PON2, TNFAIP3, ADIPOR2, F3, NEUROD1, IGF1, PTEN, SERPINE1, PPARA, SOCS1, SOCS3, MAPK1, JAZF1, MAPK8, PRKAA1, APPL1, HIPK3, EYA1, CBLB, ITCH, UCP3, GFPT1
	Obesity	ESR1, CLOCK, PIK3CA, ABCA1, IGFBP3, LDLR, HPRT1, ADIPOR2, CAST, ABHD5, IGF1, PTEN, TUB, SERPINE1, PPARA, SOCS3, UCP3, GFPT1
	Dyslipidemia	ABCA1, LDLR
	Inflammation	GJA1, TNFAIP3, PTEN, SERPINE1, SOCS1, MAPK14, TBK1, TEK, TNIP1
miR-140-3p	Diabetes	CXCL8, TAB2, HIPK3, SIRT1, SIM1, BCL2
	Obesity	AFF4, BSN, SIRT1, SIM1
	Inflammation	CXCL8, SIRT1
miR-532-3p	Diabetes	PAPPA, PEA15, GLP1R
	Obesity	PRDM16, KMT2A, GLP1R
miR-29b-3p	Diabetes	FBN1, ADAMTS9, IGF1, PTEN, VEGFA, COL4A4, SGK1, FAM167A, PPP1R15B, TNFRSF1A, SIRT1, NOTCH2, LPL, CNR1, JAZF1, TNFAIP3, PIK3R1, AKT2
	Obesity	DNMT3A, NSD1, IGF1, PTEN, STX16 SLC6A14, SIRT1, LPL, CNR1, HBEGF, PIK3R1, AKT2, CLOCK
	Dyslipidemia	LPL
	Inflammation	COL1A1, PTEN, VEGFA, TNFRSF1A, SIRT1, TNFAIP3, OTULIN
	Steatosis	AKT2
miR-146b-5p	Diabetes	APPL1, HIPK3, CCK, CCL5, CYP27B1
	Obesity	CCK, HDAC8, CYP27B1
	Inflammation	IRAK1, CCL5
miR-374a-5p	Diabetes	IGFBP3, BMP2, NKX2-2, CCL2, PDCD1, FABP2, SUMO4, ANLN, HGF, HSPA4, GLO1, NCOA1, LPL, TNFAIP3, LEPR, SELE, PAPPA
	Obesity	IGFBP3, BMP2, CCL2, FABP2, TTC8, PDE4D, NCOA1, MYT1L, ABHD5, LPL, LEPR
	Dyslipidemia	LPL
	Inflammation	IL22, CCL2, PDCD1, TNFAIP3, SELE

**Table 9:** Experimentally validated miRNAs from miRTarBase (https://mirtarbase.cuhk.edu.cn/~miRTarBase/miRTarBase_2025, accessed on 17 February 2026).

miRNA	Gene Acronym	Full Gene Name	miRTarBase (Validated)	Evidence Type	Experimental Method
miR-26a-5p	*PTEN*	phosphatase and tensin homolog	Yes	Strong evidence	Reporter assay, Western blot, qPCR
	*GSK3B*	glycogen synthase kinase 3 beta	Yes	Strong evidence	Reporter assay, Western blot, qPCR
	*HGF*	hepatocyte growth factor	Yes	Strong evidence	Reporter assay, Western blot, qPCR
	*HMGA1*	high mobility group AT-hook 1	Yes	Strong evidence	Reporter assay, Western blot, qPCR
	*ESR1*	estrogen receptor 1	Yes	Strong evidence	Reporter assay
	*ATM*	ATM serine/threonine kinase	Yes	Strong evidence	Reporter assay, Western blot, qPCR
	*ADAM17*	ADAM metallopeptidase domain 17	Yes	Strong evidence	Reporter assay, Western blot, qPCR
miR-222-5p	*ESR1*	estrogen receptor 1	Yes	Strong evidence	Reporter assay, Western blot, qPCR
miR-146a-5p	*CCL5*	C-C motif chemokine ligand 5	Yes	Strong evidence	Reporter assay
miR-142-3p	*TAB2*	TGF-beta activated kinase 1 (MAP3K7) binding protein 2	Yes	Strong evidence	Reporter assay, Western blot
miR-19a-3p	*ESR1*	estrogen receptor 1	Yes	Strong evidence	Reporter assay, Western blot, qPCR
	*ABCA1*	ATP-binding cassette subfamily A member 1	Yes	Strong evidence	Reporter assay
	*PTEN*	phosphatase and tensin homolog	Yes	Strong evidence	Reporter assay, Western blot, qPCR
	*SOCS1*	suppressor of cytokine signaling 1	Yes	Strong evidence	Reporter assay, Western blot, qPCR
	*SOCS3*	suppressor of cytokine signaling 3	Yes	Strong evidence	Reporter assay
miR-18b-5p	*ESR1*	estrogen receptor 1	Yes	Strong evidence	Reporter assay
miR-19b-3p	*ESR1*	estrogen receptor 1	Yes	Strong evidence	Reporter assay, Western blot, qPCR
	*IGF1*	insulin-like growth factor 1	Yes	Strong evidence	qPCR
	*PTEN*	phosphatase and tensin homolog	Yes	Strong evidence	Western blot
	*SOCS1*	suppressor of cytokine signaling 1	Yes	Strong evidence	Reporter assay, Western blot
	*PRKAA1*	protein kinase AMP-activated catalytic subunit alpha 1	Yes	Strong evidence	Reporter assay
	*HIPK3*	homeodomain interacting protein kinase 3	Yes	Strong evidence	Reporter assay
	*CLOCK*	clock circadian regulator	Yes	Strong evidence	Reporter assay, qPCR
miR-140-3p	*BCL2*	BCL2, apoptosis regulator	Yes	Strong evidence	Reporter assay, Western blot
miR-29b-3p	*FBN1*	fibrillin 1	Yes	Strong evidence	Reporter assay
	*PTEN*	phosphatase and tensin homolog	Yes	Strong evidence	Reporter assay
	*NOTCH2*	membrane integral NOTCH2 associated receptor 1	Yes	Strong evidence	Reporter assay, Western blot, qPCR
	*PIK3R1*	phosphoinositide-3-kinase regulatory subunit 1	Yes	Strong evidence	Western blot
	*AKT2*	AKT serine/threonine kinase 2	Yes	Strong evidence	Reporter assay, Western blot, qPCR
	*DNMT3A*	DNA methyltransferase 3 alpha	Yes	Strong evidence	Reporter assay, Western blot, qPCR
miR-146b-5p	*IRAK1*	interleukin 1 receptor associated kinase 1	Yes	Strong evidence	Reporter assay, Western blot, qPCR

## Data Availability

The original contributions presented in this study are included in the article. Further inquiries can be directed to the corresponding author.
